# Machine learning clinical decision support for interdisciplinary multimodal chronic musculoskeletal pain treatment

**DOI:** 10.3389/fpain.2023.1177070

**Published:** 2023-05-09

**Authors:** Fredrick Zmudzki, Rob J. E. M. Smeets

**Affiliations:** ^1^ Époque Consulting, Sydney, NSW, Australia; ^2^ Social Policy Research Centre, University of New South Wales, Sydney, NSW, Australia; ^3^ Department of Rehabilitation Medicine, Care and Public Health Research Institute (CAPHRI), Faculty of Health, Life Sciences and Medicine, Maastricht University, Maastricht, Netherlands; ^4^ CIR Rehabilitation, Eindhoven, Netherlands; ^5^ Pain in Motion International Research Group (PiM), Brussels, Belgium

**Keywords:** chronic pain, musculoskeletal pain, machine learning, interdisciplinary care, clinical decision support, prognosis, outcome

## Abstract

**Introduction:**

Chronic musculoskeletal pain is a prevalent condition impacting around 20% of people globally; resulting in patients living with pain, fatigue, restricted social and employment capacity, and reduced quality of life. Interdisciplinary multimodal pain treatment programs have been shown to provide positive outcomes by supporting patients modify their behavior and improve pain management through focusing attention on specific patient valued goals rather than fighting pain.

**Methods:**

Given the complex nature of chronic pain there is no single clinical measure to assess outcomes from multimodal pain programs. Using Centre for Integral Rehabilitation data from 2019–2021 (*n* = 2,364), we developed a multidimensional machine learning framework of 13 outcome measures across 5 clinically relevant domains including activity/disability, pain, fatigue, coping and quality of life. Machine learning models for each endpoint were separately trained using the most important 30 of 55 demographic and baseline variables based on minimum redundancy maximum relevance feature selection. Five-fold cross validation identified best performing algorithms which were rerun on deidentified source data to verify prognostic accuracy.

**Results:**

Individual algorithm performance ranged from 0.49 to 0.65 AUC reflecting characteristic outcome variation across patients, and unbalanced training data with high positive proportions of up to 86% for some measures. As expected, no single outcome provided a reliable indicator, however the complete set of algorithms established a stratified prognostic patient profile. Patient level validation achieved consistent prognostic assessment of outcomes for 75.3% of the study group (*n* = 1,953). Clinician review of a sample of predicted negative patients (*n* = 81) independently confirmed algorithm accuracy and suggests the prognostic profile is potentially valuable for patient selection and goal setting.

**Discussion:**

These results indicate that although no single algorithm was individually conclusive, the complete stratified profile consistently identified patient outcomes. Our predictive profile provides promising positive contribution for clinicians and patients to assist with personalized assessment and goal setting, program engagement and improved patient outcomes.

## 1. Introduction

Chronic musculoskeletal pain (CMP) is a prevalent condition impacting 1.7 billion people globally and is the biggest contributor (17%) to years lived with disability worldwide (1). Patients experience debilitating pain, fatigue, and limited mobility, causing reduced social activity, employment, and quality of life. Patients with chronic pain often suffer long term where all available interventions such as nerve blocks, corticosteroid injections, spondylodesis, or pharmacological treatments have been ineffective. Interdisciplinary Multimodal Pain Treatment (IMPT) programs have been shown to provide positive and sustained outcomes where all other modalities have failed (2–4). The Centre for Integral Rehabilitation (CIR), a pain rehabilitation clinic with seven locations in the Netherlands, provides a 10-week IMPT program for patients with CMP reporting promising positive outcomes (3, 5, 6). The CIR program supports CMP patients in modifying their behavior and assists with pain management by focusing patient attention on specific value-based goals rather than fighting pain.

As patients referred to IMPT programs have exhausted all pain management pathways, any minimal clinically important outcome is notable, and improvements above 50% of patients treated at CIR are remarkable. The CMP study group indicates particularly high proportions of patient improvement across some outcome dimensions including disability, where 85% of patients reported improvement for the General Perceived Effect (GPE) disability measure, pain, with improved GPE pain scores for 69% of patients, and coping, where 86% of patients reported an improved GPE coping outcome. Similar positive IMPT outcomes have been reported and have shown sustained improvements in the long term (2, 7–9).

Despite these positive outcomes, CMP is a complex multidimensional condition with no single consistently reliable endpoint and characteristic variation in outcome measures. For this reason, we defined a framework of 10 clinical endpoints and developed a corresponding profile of algorithms to assess new patients referred for IMPT. Collectively, the framework of machine learning models was used to develop a predictive multidimensional patient profile to assist clinicians with patient selection and individual goal-setting. The primary study question was whether machine learning could assist clinical decision support when assessing CMP patients for the IMPT program.

There are numerous examples of machine learning being successfully implemented in clinical settings, including for diabetes (10), prediction of low back pain (11), improvement of back pain outcomes (12), self-referral decisions (13), and self-management of low back pain (14). Machine learning methods have been used to assess pain diagnosis and prediction of developing chronic pain (15). These examples relate to earlier diagnostic phases including imaging, where machine learning methods have been shown to be well suited. Our patient study group, by comparison, has all previously been diagnosed with long-term CMP. We were not examining chronic pain diagnosis but whether machine learning methods could contribute to the prognostic identification of patients most likely to achieve positive outcomes from the IMPT program. This is the first research to our knowledge aimed at developing a machine learning decision support framework for an IMPT program.

Our research project also considered how patient prognostic algorithms could be implemented into IMPT practice. Although machine learning methods have developed rapidly in healthcare, they have been slow to enter clinical practice due to complex “blackbox” abstraction and the need for thorough validation prior to implementation (16). Evidence suggests that these advancing methods have substantial potential to contribute to individualized care, particularly if stratified for patient risk, so clinicians can consider and avoid harm (17). In this context, we have used commonly established classes of algorithms and considered a recently developed clinician checklist of questions to assist assessment of algorithm development, data quality, validation and performance, risks, and ethical concerns (18). An implicit focus of our research was to investigate how machine learning models can assist clinical decision support to further improve the patient outcome and IMPT program efficiency. Although preliminary research indicates that the CIR program is likely cost-effective (2), a predictive multidimensional patient profile may assist clinicians and patients with assessment, program engagement, and goal-setting and further contribute to program effectiveness and related cost-effectiveness.

## 2. Methods

**Figure 1:**
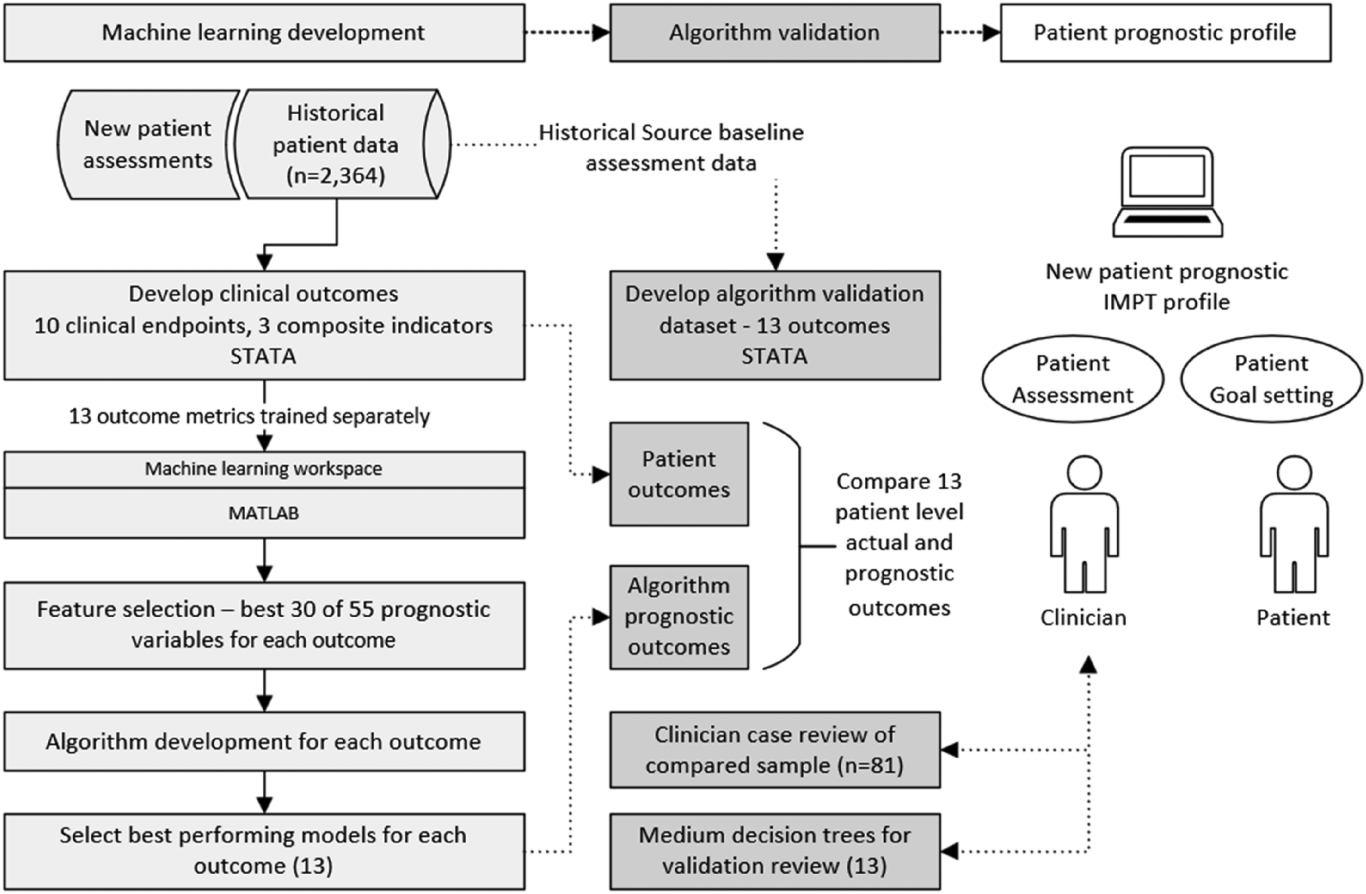
Diagram of methods, machine learning development, and algorithm validation process. Algorithm development was undertaken for multiple classification models including decision trees, discriminant analysis, support vector machine (SVM), logistic regression, nearest neighbor, naive Bayes, kernel approximation, ensembles, and neural networks. The best-performing model was selected for each of the 13 outcome measures.

The study developed a framework of clinical endpoints to assess the complex variation in outcomes reported by patients participating in the CIR IMPT program. As there is no single reliable IMPT outcome measure relevant for all patients, 10 clinical measures and 3 composite metrics were defined to assess patient outcomes and provide the basis for supervised machine learning across demographic and potential prognostic baseline variables. The approach developed sequential stages of data preprocessing, selecting data items with the highest prognostic performance, training each machine learning model, and validating algorithm accuracy against source patient data, see Figure 1. In addition to patient-level validation of each of the 13 algorithms, compared to source data actual outcomes, an independent clinician case file review was undertaken for a targeted subgroup of patients, intentionally based on challenging patient assessments where most predictive indicators were negative (*n* = 81). This supplementary validation process aimed to further verify algorithm accuracy and examine the potential value of having the prognostic profile when assessing new IMPT patients.

The initial phase defined the framework of outcome measures based on clinician advice. Each outcome metric was derived in the patient study dataset to provide endpoints for the supervised machine learning development. All 13 algorithms were then separately trained across multiple classification models. This provided two complete sets of algorithms; first, the best-performing models for each of the 13 outcomes were used as the primary prognostic models. Second, although decision tree models generally achieve slightly lower accuracy, they provide a visual structure of threshold values across predictive variables. For this reason, a complete second set of medium decision tree algorithms was prepared for each of the 13 outcome measures for supplementary algorithm validation and enhanced transparency for clinician review of decision branch thresholds.

### 2.1. Patient study group

The IMPT study data include all patients accepted to the CIR program over 3 years from 2019 to 2021 who completed the full 10-week intervention (*n* = 2,364). A high proportion of patients were women (72.5%), consistent with IMPT populations in the Netherlands (19). The mean (±SD) patient age was 43.8 ± 13.0 years. All patients were previously diagnosed with long-term CMP and referred to the IMPT program following comprehensive pain management investigations, typically over multiple years. As study group patients have exhausted all pharmacological or clinical interventions, any IMPT minimal clinically important change for this cohort is considered significant.

### 2.2. CIR IMPT program

The CIR program comprises a treatment team of physiotherapists, psychologists, and a physiatrist. The IMPT program aims to improve the daily functioning, participation, and quality of life of patients with CMP using a combination of physical and psychosocial treatment methods, including emotional awareness and expression therapy, pain neuroscience education, acceptance and commitment therapy, graded activity, exposure *in vivo*, and experiential learning through physical training (5). Treatment phases cover 10-week participation in the program including a start phase in week 1 (T0), an education phase in weeks 2–3, a skills learning phase during weeks 4–10, a mid-trajectory evaluation in week 5 (T1), and a final evaluation in week 10 (T2). A subgroup of patients (around 12%) are subsequently referred for further second treatment of 5–20 h, following a 6-week break, with a final follow-up data point (T3).

The machine learning development is based on outcomes between baseline at the entry to the program (T0) and the change in each outcome measure at the completion of the full 10 weeks (T2). This is the most effective timeframe to assess most outcomes, as patients generally respond to treatment by T2, although further improvements are reported in patients approved for extended second treatment (T3). Further longitudinal data points are reported at 3 months (T4), 6 months (T5), and 12 months (T6). The current study did not assess timepoints beyond T2.

### 2.3. Data source and preprocessing

The patient dataset is collected by CIR for all patients with chronic pain referred to the IMPT program, maintained by Asterisque (a software developer), and validated by Maastricht University, Department of Rehabilitation Medicine. New patients complete an online survey of baseline demographic and questionnaire data at timepoint T0. There were variations in sample sizes for each outcome due to the available study group at timepoint T2 or missing data for patients not collected in the initial study phases. The endpoints were developed as binary indicators where one indicates a positive outcome when the defined rule is met; otherwise, each outcome was defined as zero. Data analysis and preprocessing were undertaken in STATA V16.1. (StataCorp LP, College Station, TX, United States). All records in the patient dataset were deidentified with a unique patient number. Medical Research Ethics Committee Isala Zwolle reviewed this study (case number assigned: 200510), and as all patients have consented to the use of their data for research, further ethics approval was not required.

### 2.4. Patient outcomes

CMP is a complex condition, and patient responses to the IMPT program are often subjective and highly variable. There is no clear clinical endpoint to reliably indicate a positive program outcome. Some patients report positive improvements in one domain and not in others. For this reason, we defined a set of 10 clinical endpoints across five clinically relevant domains, in line with the complex assessment clinicians undertake with these patients, see Table 1. The domains include (1) activity/disability, (2) pain, (3) fatigue, (4) coping, and (5) quality of life (19). Outcome measurement instruments are grouped within each domain by the measure domain group number. Categories 1, 2, and 3 are summarized by a group indicator defined as any one of the endpoints within the group, denoted C1, C2, and C3, respectively. This was included to reflect clinician practice where a positive outcome in any one of the indicators within each group is considered a positive result. The combined outcome framework includes 10 separate clinical endpoints and 3 composite summary indicators. Further details of the clinical outcome endpoints, including measurement constructs and questionnaire response scales, are provided in Supplementary Table 1.

**Table 1:** Description of clinical measures and endpoint metrics.

Measure domain group number	Measurement instrument	Measurement domain	Measurement score	Measurement metric definition
1.1	PDI (20)	*Disability*	A sum score is calculated, ranging from 0 to 70. Lower scores mean lower disability	A decrease of 9 or more points (21)
1.2	GPE disability <4	*Disability*	Lower scores mean more improvement	Patients with scores 1, 2, or 3 are considered responders (22)
1.2a	GPE disability <3	*Disability*	Lower scores mean more improvement	Patients with scores 1 or 2 are considered responders (22)
1.3	PSC (23, 24)	*Disability/activity*	The average of three VAS scores is calculated	Effect size Cohen >0.8 (25)
C1	Measure 1.1 or 1.2 or 1.3	*As above*	A positive outcome if any group 1 measures are positive	Group 1 as above
2.1	GPE pain	*Pain*	Lower scores mean more improvement	Patients with scores 1, 2, or 3 are considered responders (22)
2.2	NRS pain last week	*Pain*	Lower scores mean less pain	Reduction of 30% compared to the baseline (26)
C2	Measure 2.1 or 2.2	*As above*	A positive outcome if any group 2 measures are positive	Group 2 as above
3.1	NRS fatigue last week	*Fatigue*	Lower scores mean less fatigue	Reduction of 30% compared to the baseline (26)
3.2	CIS (27)	*Fatigue*	A sum score is calculated, ranging from 20 to 140. Lower scores mean less exhaustion	Effect size Cohen >0.8 (25)
C3	Measure 3.1 or 3.2	*As above*	A positive outcome if any group 3 measures are positive	Group 3 as above
4	GPE coping	*Coping*	Lower scores mean more improvement	Patients with scores 1, 2, or 3 are considered responders (22)
5	SF-12 PCS (28)	*Health-related quality of life physical component score*	A physical (PCS) component is calculated from weighted responses. The summary score ranges from 0 to 100. Higher scores mean better physical health	Effect size Cohen >0.8 (25)

PDI, pain disability index; GPE, general perceived effect; PSC, patient-specific complaints; NRS, numeric rating scale; CIS, checklist individual strength; SF-12 PCS, short-form quality of life survey physical component score; VAS, visual analogue scale.

GPE <3 indicates totally or much improved; GPE <4 indicates totally, much, or somewhat improved.

Each endpoint is defined by either an absolute change in score, a range of categorical outcomes, a percentage improvement, or the effect size as measured by Cohen's *d*. Cohen's threshold was defined as a large effect size of 0.8, as small and medium values resulted in higher proportions of positive outcomes, which were less balanced for algorithm training (25). Data analyses were undertaken on mixed categorical and continuous variables, with algorithm training based on derived binary outcomes indicating improved (positive) or unimproved (negative) endpoint calculations. The outcome thresholds are based on clinician assessment practice, and some are described in existing literature, as presented in Table 1.

A substantial proportion of study group patients attained individual positive outcome measures ranging from 36.6% for fatigue during the last week to 86.3% for GPE coping. Most of the endpoints indicate a positive program outcome, with 7 of the 10 measures achieved by more than 50% of patients. Additional supplementary endpoints were examined in which the proportion of positive outcomes was high to provide more balanced training data and assess potential classification bias in samples. For example, algorithm 1.2 GPE disability <4 is defined as a survey response of “totally,” “much,” or “somewhat” improved, with 84.9% of patients meeting this threshold. The additional algorithm 1.2a GPE disability <3 established a higher threshold in which the response was reduced to “totally” or “much” improved, providing a smaller 39.7% of positive patients. Both measures represented different levels of a positive outcome and were included to examine algorithm accuracy.

### 2.5. Prognostic variables and feature selection

From a review of the patient dataset, 55 demographic and potential prognostic variables were identified. All patient variables considered to have potential prognostic value were included for feature selection analysis to assess the most valuable data items of each endpoint. Patient string variables stored as text, such as patient comments, were highly variable and therefore excluded as prognostic candidates. Most prognostic data items were used for all clinical endpoints, with additional supplementary data items used in clinician-selected measures, consistent with the World Health Organization model for rehabilitation. For example, for the quality-of-life endpoint using the Short-Form (SF-12) physical component score, pain disability index (PDI) and patient-specific complaints (PSC) were added as prognostic factors as these are from different outcome dimensions. This optimizes prognostic factors by eliminating duplication of variables from dimensions related to the endpoint, reducing covariation. Details of specific prognostic variables used for each outcome measure are provided in Supplementary Table 2.

From the 55 prognostic candidate variables, feature selection algorithms were conducted separately for each clinical endpoint using the MATLAB Statistics and Machine Learning Toolbox (Release 2022b) to rank variables by predictive importance. Feature selection has been shown to reduce model overfitting and improve model accuracy and performance (29). Feature selection testing was undertaken to assess multiple methods including minimum redundancy maximum relevance, chi-squared, ANOVA, and Kruskal–Wallis estimates. The resulting feature selection was consistent across methods, and the minimum redundancy maximum relevance algorithm was selected as the standard used for all analyses across outcomes (30). Predictive variables ranked lower than 30 had very low predictive power, and results improved slightly when variables below 30 were excluded. Details of feature selection rankings for each endpoint are provided in Supplementary Table 3, with predictor importance scores for each prognostic variable. Prognostic variable descriptions and summary figures for the number of times features selected across all 13 outcomes are provided in Supplementary Table 4.

### 2.6. Machine learning methodology

As patient profiles reflect variations in the defined outcome measures, all 13 algorithms were trained individually as supervised classification models. Each clinical endpoint response variable was then used to train multiple classifier algorithms including decision trees, discriminant analysis, support vector machines, logistic regression, nearest neighbors, naive Bayes, kernel approximation, ensembles, and neural networks. Each clinical endpoint was separately trained with the selected best 30 predictive variables for that individual outcome using the binary response variable indicating an improvement in that endpoint or not. All models were trained and cross-validated using five folds of patient data to help avoid overfitting and estimate predictive accuracy. This randomly divides the data into five equal partitions, trains each algorithm on a training fold, and cross-validates against validation folds to calculate the average validation error and provide an estimate of predictive accuracy. The model for each outcome measure with the highest accuracy score was selected as the best-performing algorithm. These best-performing machine learning models provide the clinician and patient with each outcome measure across the stratified prognostic profile, with a simple majority (positive or negative) summary measure to gauge overall results.

Machine learning models are commonly considered to lack transparency in how they produce results. To investigate potential decision logic, we developed a second set of algorithms using a medium classification tree model for each of the 13 measures. Classification trees can be generated as graphical figures showing the decision rules of a classification problem, in our case, the pathway to classify a positive outcome measure. Patient study data preprocessing, outcome variable specification, and calculations were undertaken in STATA V16.1. (StataCorp LP, College Station, TX, United States). Final summary data of the prognostic variables for each outcome were individually extracted and exported to MATLAB R2022a for machine learning analyses.

### 2.7. Statistical analysis

To assess model classification performance of each outcome algorithm, a confusion matrix was calculated to examine true positive rates (TPRs) and false negative rates (FNRs). Model performance was calculated using the receiver operating characteristic (ROC) area under the curve (AUC), accuracy (number of correct predictions/total predictions), recall [true positive rate (TPR)], specificity [true negative rate (TNR)], precision [positive predictive value (PPV)], and F1 scores (harmonic mean of precision and recall). The AUC curves calculate the TPR compared to the FPR for different thresholds of classification scores to assess the overall quality of each model. Although there is no universally accepted threshold for AUC prognostic significance, AUC scores between 0.6 and 0.7 are considered acceptable and between 0.5 and 0.6 are viewed as poor, with 0.5 representing no prognostic value or equivalent to a random guess (31).

### 2.8. Algorithm validation

An algorithm validation framework was developed using deidentified baseline data from the patient dataset. Baseline prognostic variables were re-extracted, and each algorithm model was conducted to derive the predicted outcome for each patient in each of the 13 outcome measures. This provided patient-level validation of the prognostic accuracy of each algorithm compared to the derived actual outcomes study group patients achieved. Further metrics were calculated to verify each outcome measure per patient and establish the number of the 13 outcomes that were correctly validated. In addition to patient-level cross validation, a supplementary sample of 81 patients recording mostly negative outcomes was independently clinician-reviewed. This additional validation was undertaken to verify predictive results and investigate whether having the prognostic profile at baseline assessment was potentially valuable for individual patient selection and goal-setting.

There are two prognostic validation elements produced through the machine learning models: First, the complete set of individual prognostic outcomes is generated for clinician assessment of new program patients. This includes accuracy metrics for each outcome measure to indicate low or high true positive or true negative models. Additionally, a simple summary indicator is provided from the count of positive versus negative prognostic outcomes to indicate a positive or negative majority. This is not presented as a prescriptive indicator but a basic grouping in which multiple measures consistently point to a positive or negative patient outcome.

## 3. Results

Study group characteristics show a high proportion of female patients (72.5%), see Table 2. The mean (±SD) patient age was 43.8 ± 13.0 years, with 35.9% over 50 and 11.7% over 60 years of age. All patients referred to the CIR IMPT have been diagnosed with CMP and classified through the Working Group on Pain Rehabilitation in the Netherlands (Werkgroep Pijnrevalidatie Nederland, WPN). Based on this rating of the complexity of pain symptoms, the majority (85%) were assessed as WPN 3 chronic pain syndrome and 14% as WPN 4 (maximal score), showing a clear indication of psychosocial factors underlying and maintaining pain and its associated disability. Based on the body mass index (BMI), most patients were classified as overweight (65.7%, BMI ≥ 25) and 30.4% met the threshold considered obese (BMI ≥ 30), including 8.2% obesity class II (BMI ≥ 35) and 3.5% obesity class III (BMI ≥ 40).

**Table 2:** Study group demographic and baseline characteristics (*n* = 2,364).

	%	*n*
Gender		
Male	27.5	649
Female	72.5	1,715
Age group (years)		
<20 years	1.4	33
20–29	15.3	361
30–39	21.7	513
40–49	25.8	610
50–59	24.2	571
60 and over	11.7	276
Pain diagnosis		
WPN 2 chronic pain syndrome	0.3	7
WPN 3 chronic pain syndrome	84.6	1,998
WPN 4 chronic pain syndrome	14.0	331
Other pain syndrome	0.9	21
Psychiatric disease	0.2	4
BMI group (kg/m^2^)		
Less than 20	4.3	92
20–24.9	30.0	643
25–29.9	35.3	757
30–34.9	18.7	402
35–39.9	8.2	175
40 or over	3.5	76
Number of pain locations		
1	10.2	231
2–5	51.0	1,159
>5	38.9	885
Living status		
Alone	17.5	397
With partner	65.4	1,486
Living apart together	5.2	119
With parent(s)	6.5	148
Other	5.3	121
Highest level of education		
No	0.6	14
Primary school	2.8	63
Pre-vocational secondary	13.8	312
Secondary vocational	44.2	1,003
Senior general secondary/Pre-university	7.1	162
Senior general secondary/Pre-university not finished	6.4	146
Higher professional/University	23.7	537
Postdoctoral	1.4	31
Paid employment		
No	36.1	819
Yes	63.9	1,450
Duration of symptoms		
0–3 months	1.1	24
3–6 months	6.3	144
6–12 months	13.1	298
1–2 years	17.0	386
2–5 years	24.7	561
More than 5 years	37.8	860
Use of pain medication		
No	37.3	848
Yes	62.7	1,423
Baseline scores	Mean (SD)	*n*
PDI	38.7 (12.3)	2,294
PSC	71.3 (13.7)	1,200
NRS pain	6.5 (1.8)	2,273
NRS fatigue	7.2 (2.0)	2,272
CIS	97.3 (22.2)	2,254
SF-12 PCS	30.9 (6.7)	2,284

WPN, classification of the Working Group on Pain Rehabilitation Netherlands (Werkgroep Pijnrevalidatie Nederland) (32); BMI, body mass index; PDI, pain disability index; PSC, patient-specific complaints; NRS, numeric rating scale; CIS, checklist individual strength; SF-12 PCS, short-form quality of life survey physical component score.

Minor variations in subgroup sample sizes were due to missing data. PSC was introduced in 2020.

Most patients experienced pain in multiple anatomical locations, with 89.9% reporting more than one and 38.9% indicating more than five locations. Most patients (63.9%) also reported being in paid employment, and 62.7% were using pain medication.

### 3.1. Feature selection

The feature selection minimum redundancy maximum relevance algorithm was conducted for each clinical endpoint, and the 30 highest-ranking features were selected for each classification model training. There is a minor variation in the number of features across endpoints from 49 to 55, reflecting diagnostic dimensions. Tests were undertaken for comparison without feature selection using all 55 variables, and other feature selection methods were also assessed and produced consistent results. Reducing to the 30 most significant features for each endpoint increased accuracy slightly and was more efficient. Details of prognostic variables used for each outcome measure are provided in Supplementary Table 2. Details of all 55 prognostic variables and the 30 selected for each model are provided with minimum redundancy maximum relevance ranking scores in Supplementary Table 3.

Although there is characteristic variation in the most predictive features across each endpoint, three baseline prognostic variables were selected for all models: (1) treatment control (How much do you think your treatment can help your illness? Brief Illness Perception Questionnaire), (2) worst pain last week (NRS), and (3) disability pension/sick leave (response is no or five different yes options). A further five variables were prominent and used in 92% (12 of the 13 models): (1) CIR location, (2) cognitive fusion (Psychological Inflexibility in Pain Scale), (3) timeline (How long do you think your illness will continue?), (4) coherence (How much does your illness affect you emotionally? Brief Illness Perception Questionnaire), and (5) depression (Hospital Anxiety and Depression Scale (HADS)). Other prominent prognostic factors were selected in over 75% (10 of 13) of models, including hours per week of paid work, level of education, duration of pain (6 categories), pain diagnosis, home adaptations, time willing to spend on treatment, 6-min walking distance, and self-reported work capacity (NRS 0–10). Age and gender were selected in around half of the trained models, while patient BMI was not selected in any, likely due to the high proportion of the study group that was overweight, suggesting that BMI was not a distinguishing factor for a positive program outcome. This shows that feature selection helps adjust for imbalanced prognostic variables such as the high proportion of female or overweight patients. Summary details of selected variables across all 13 outcome models are provided in Supplementary Table 4.

### 3.2. What is a positive IMPT patient outcome?

As referral to the program is a final pathway where all other therapies have failed, a positive outcome for any patient in this cohort is notable. The high proportion of study group patients achieving a positive result is exceptional, ranging from around 40% to above 85% across the 13 outcome measures, see Table 3. The high proportion of positive outcomes in the training data results in similarly high levels of predicted positive outcomes. This is not a machine learning weakness but due to the imbalanced proportion of positive or negative outcomes, as reflected in the corresponding true positive and true negative rates. As expected, the algorithms replicate and generally amplify the imbalanced training data so that high proportions of patients with a positive outcome measure result in equally or higher predicted true positive rates. Patient-level validation of positive and negative algorithm results shows that the proportions of each outcome are consistent with estimated true positive and true negative rates, see Supplementary Table 5. In practice, the prognostic profile results will present algorithm accuracy figures for clinician reference. In summary, the results show that although there is expected variation in each outcome measure, the algorithms establish prognostic indicators which collectively build a stratified positive or negative patient assessment profile.

**Table 3:** CMP machine learning pilot—algorithm results.

	Outcome measure (algorithm type)	*N*	% Positive	AUC	Accuracy	Recall TPR	Specificity TNR	Precision PPV	F1 score
1.1	PDI (ENS-SD)	2,002	58.2	0.63	0.61	0.89	0.23	0.61	0.73
1.2	GPE disability <4 (ENS-BT)	1,657	84.9	0.53	0.85	1.00	n/a	0.85	0.92
1.2a	GPE disability <3 (SVM-L)	1,657	39.7	0.65	0.63	0.32	0.83	0.56	0.41
1.3	PSC (SVM-CG)	990	70.1	0.49	0.70	1.00	n/a	0.70	0.82
C1	1.1 or 1.2 or 1.3 (ENS-SD)	2,036	84.9	0.64	0.85	1.00	n/a	0.85	0.92
2.1	GPE pain (ENS-BT)	1,654	68.7	0.62	0.69	0.97	0.09	0.70	0.81
2.2	Pain NRS (LR)	1,645	42.4	0.65	0.62	0.37	0.81	0.59	0.45
C2	2.1 or 2.2 (ENS-BT)	1,655	72.6	0.60	0.73	0.98	0.06	0.73	0.84
3.1	Fatigue NRS (ENS-SD)	1,642	36.3	0.61	0.64	0.09	0.96	0.56	0.15
3.2	CIS total (ENS-SD)	1,949	52.3	0.60	0.57	0.72	0.42	0.57	0.64
C3	3.1 or 3.2 (SVM-FG)	1,788	61.5	0.52	0.61	1.00	n/a	0.61	0.76
4	GPE coping (LR)		86.3	0.64	0.86	1.00	0.02	0.86	0.93
5	SF-12 PCS (KNB)		59.7	0.58	0.60	0.94	0.10	0.61	0.74

CMP, chronic musculoskeletal pain; AUC, area under the curve; TPR, true positive rate; TNR, true negative rate; PPV, positive predictive value; machine learning endpoint; PDI, pain disability index; GPE, global perceived effect; PSC, patient-specific complaints; NRS, numeric rating scale; CIS, checklist individual strength; SF-12 PCS, short-form quality of life survey physical component scale. Machine learning model in brackets: ENS-SD, ensemble subspace discriminant, ENS-BT, ensemble boosted trees; SVM-L, support vector machine linear; SVM-CG, support vector machine coarse Gaussian; LR, logistic regression; SVM-FG, support vector machine fine Gaussian; KNB, kernel naive Bayes. n/a, not applicable due to imbalanced endpoint data.

Results are based on the highest estimated accuracy across all tested algorithms.

### 3.3. Performance of the machine learning models

As expected, no individual clinical measure produced a single reliable prognostic indicator to consistently predict a positive program outcome, see Table 3. This is in line with the complex multidimensional clinician assessment process, which does not rely on a single baseline patient questionnaire but uses each prognostic indicator to develop a patient profile. Similarly, our framework of machine learning indicators was not anticipated to produce a single prescriptive measure. The algorithm-estimated accuracy scores reflect the high variation in individual outcomes, with 9 of the 13 measures within the indicative AUC range between 0.6 and 0.7 considered “acceptable” and the remaining 4 models below 0.6 and viewed as having “poor” prognostic discrimination.

### 3.4. Classification decision trees and algorithm transparency

The second set of developed algorithms used a medium classification tree model for each of the 13 outcome measures to generate graphical figures showing the decision rules and threshold values at each branch. The model accuracy scores were marginally lower than the best-performing algorithms, but the results produced similar patterns for estimated true positive and true negative rates, see Table 4. The decision trees were clinician-reviewed to assess branch structures and leaf node thresholds. Overall, the classification trees did not provide further insight into how results were derived. This is because the branches establish multiple combinations of decision point values. Although it was possible to follow a rule pathway and obtain a positive or negative result, the branches were contingent on multiple tree levels and did not provide generalized useful threshold values for individual outcome measures. The classification trees did, however, provide a supplementary view of combinations of prognostic variables. For example, decision branches showed positive outcome threshold combinations for high anxiety and low depression scores, see Supplementary Image 1. The decision tree shows the algorithm path to a positive outcome of a baseline HADS anxiety score of 12 (which is indicative of a heightened level of anxiety) combined with a HADS depression score of <6 (not indicative of any sign of depression). This is not a common profile and could make the clinician aware that addressing specific fears by using exposure *in vivo* could be highly relevant. These types of secondary associations could provide supplementary insights to tailor further algorithms or develop further composite metrics for the basis of ongoing reinforced machine learning.

**Table 4:** CMP machine learning pilot—algorithm results—supplementary medium classification trees.

	Outcome measure	% Positive	AUC	Accuracy	Recall TPR	Specificity TNR	Precision PPV	F1 score
1.1	PDI	58.2	0.57	0.59	0.86	0.21	0.60	0.71
1.2	GPE disability <4	84.9	0.57	0.83	0.97	0.02	0.85	0.91
1.2a	GPE disability <3	39.7	0.60	0.59	0.36	0.73	0.47	0.41
1.3	PSC	70.1	0.58	0.66	0.90	0.10	0.70	0.79
C1	1.1 or 1.2 or 1.3	84.9	0.56	0.84	0.98	0.05	0.85	0.91
2.1	GPE pain	68.7	0.59	0.67	0.93	0.09	0.69	0.80
2.2	Pain NRS AVP3	42.4	0.56	0.55	0.37	0.69	0.47	0.41
C2	2.1 or 2.2	72.6	0.58	0.71	0.95	0.09	0.73	0.83
3.1	Fatigue NRS AVP5	36.3	0.55	0.59	0.24	0.80	0.40	0.30
3.2	CIS total	52.3	0.55	0.53	0.79	0.25	0.54	0.64
C3	3.1 or 3.2	61.5	0.56	0.58	0.87	0.12	0.61	0.72
4	GPE coping	86.3	0.60	0.85	0.97	0.05	0.87	0.92
5	SF12 PCS	59.7	0.53	0.58	0.91	0.09	0.60	0.72

CMP, chronic musculoskeletal pain; AUC, area under the curve; TPR, true positive rate; TNR, true negative rate; PPV, positive predictive value; machine learning endpoint; PDI, pain disability index; GPE, global perceived effect; PSC, patient-specific complaints; NRS, numeric rating scale; CIS, checklist individual strength; SF-12 PCS, short-form quality of life survey physical component scale.

### 3.5. Validation of patient profile of algorithms

As there was not expected to be an individual clear prognostic outcome measure, supplementary validation was undertaken to examine how the profile of algorithms would perform for new patients. We did this by re-extracting the prognostic variables from the source training data and conducting the 13 algorithm models for each patient, developing a cross validation compared with derived actual outcomes for each measure.

There is variation in the number of outcomes reported for each patient as an additional outcome measure (PSC) was introduced during the study period. To test the overall direction of the algorithms, we first developed a basic indicator where most outcomes were either positive or negative. This was derived across all patients irrespective of the number of available outcomes, for example, where seven outcomes were available for a patient, with four positive ones indicating a majority. The resulting validation per patient compares the majority positive or negative indicator for outcomes with the corresponding majority indicator for the prognostic measures. The algorithms produced predictive outcomes for all 13 models for all study group patients, whether they had reported all outcomes or not.

The actual patient outcome indicator and the separate machine learning indicator were then compared to validate which patients had a consistent assessment, see Table 5. This simple majority-based perspective of the algorithm profile validated 1,470 patients (75.3%) with the same majority indicator. Of the patients confirmed with algorithm versus actual study data outcomes, most (1,330; 90.5%) were positive majority outcomes and 9.5% were majority negative outcomes. This is consistent with the high proportion of positive patient outcomes in the training data and the resulting algorithms’ high true positive values. This provided a validation sample (*n* = 1,953) that excluded patient outcomes where an even number did not provide a majority one way or the other.

**Table 5:** Algorithm validation by the proportion of positive patient outcome measures.

Algorithm validation	Positive (majority)	Negative (majority)	Total validated	
Confirmed with study data outcome	1,330	140	1,470	75.3%
	90.5%	9.5%	100.0%	
Different to study data outcome	476	7	483	24.7%
	98.6%	1.4%	100.0%	
Total predicted positive or negative	1,806	147	1,953	100.0%
	92.5%	7.5%	100.0%	

Based on patients with a majority or minority of positive outcome measures in study group, *n* = 1,953. Positive is defined as the number of positive measures > the number of negative measures. Source patient data includes variations in the number of outcome measures due to incomplete data; patients with an even number of measures that were equally positive or negative are excluded as no defined majority indicator. Machine learning models generate a complete set of 13 prognostic measures for all patients but are only possible to validate for patients with a majority indicator in baseline data.

The initial validation did not examine which specific outcome measures were positive or negative, as it was not possible to crosscheck individual outcomes where data were not available. Most patients reported data for 12 or the complete 13 clinical endpoints (1,524; 78%), see Table 6. This shows the distribution of how many outcomes were available for the 1,470 patients validated, where most prognostic outcomes were confirmed against a corresponding majority in the source study dataset.

**Table 6:** Number of patient outcomes available in the study dataset.

Total outcomes	Algorithm validation confirmed	Different from study data outcome	Total patients	% positive	% By outcomes
1	2	3	5	40.0%	0.3%
2	4	6	10	40.0%	0.5%
3	14	8	22	63.6%	1.1%
4	80	57	137	58.4%	7.0%
5	156	24	180	86.7%	9.2%
6	8	2	10	80.0%	0.5%
7	1	0	1	100.0%	0.1%
8	8	5	13	61.5%	0.7%
9	4	1	5	80.0%	0.3%
10	16	7	23	69.6%	1.2%
11	20	3	23	87.0%	1.2%
12	426	140	566	75.3%	29.0%
13	731	227	958	76.3%	49.1%
Total	1,470	483	1,953	75.3%	100.0%

Gray shading: patient validation subgroup where complete 13 outcome data were available (*n* = 958).

The next phase of validation focused on the subgroup of patients for which 13 complete outcome data were available (*n* = 958, shaded row), see Table 6. For this subgroup, each prognostic algorithm was individually validated with the corresponding outcome from the study dataset for each patient. So, if the predicted PDI was the same for both algorithm and dataset outcomes, whether positive or negative, this was flagged as valid for each of the 13 measures. The total number of validated algorithms for each patient was then counted, providing a patient matrix with the number of positive predicted outcomes and the number of individual validated measures for the same patient, see Table 7. The top section (left side light gray area) shows negative majority outcomes of seven or more, and the right-hand section (light gray area) indicates a majority of validated outcomes of seven or more. These results, validated on individual algorithms for the subgroup with complete data, are consistent with the simple majority indicator for the full study group. The number of patients with a validated negative majority is 81 (8.5%, dark shaded section) compared to 9.5% for the full study group sample.

**Table 7:** Number of positive algorithm outcomes by the number of validated outcomes.

Count algorithm positive	Count algorithm negative	Number of validated outcomes (algorithm = actual outcome)										
		4	5	6	7	8	9	10	11	12	13	Total
1	12	0	0	0	0	0	0	0	0	2	0	2
2	11	0	0	0	0	0	0	0	0	0	0	0
3	10	0	0	0	0	0	0	1	1	0	1	3
4	9	0	0	0	0	0	2	3	3	3	0	11
5	8	0	0	0	0	2	4	4	4	6	3	23
6	7	0	0	0	2	4	5	12	9	7	3	42
7	6	0	2	12	8	7	19	17	20	5	3	93
8	5	0	5	7	9	35	36	40	37	12	4	185
9	4	3	5	2	12	14	38	39	47	24	10	194
10	3	0	2	6	8	17	25	45	54	45	18	220
11	2	0	0	0	1	1	2	8	27	25	19	83
12	1	0	0	0	0	0	1	0	12	34	25	72
13	0	0	0	0	0	0	0	0	3	7	20	30
Total		3	14	27	40	80	132	169	217	170	106	958

Patient validation subgroup where complete 13 outcome data were available (*n* = 958).

### 3.6. Clinician validation review

The final validation step was to undertake an independent clinician case file review of the validated negative sample of 81 patients. This sample was chosen as a particularly challenging subgroup to identify, given the generally high proportion of positive outcomes. The deidentified patient codes from the study dataset were provided for clinician reidentification and review of each patient. This individual verification of algorithm performance confirmed that half of the patients (40/81; 49.4%) did not achieve an improvement or insufficient improvement to be clinically significant. The remaining half of patients reported various indicative or subjective outcomes for further review and ongoing outcome measure development. Collectively, the stratified prognostic profile appears to be potentially valuable for clinician assessment, selection of new patients, and consideration of setting individual patient goals.

### 3.7. IMPT prognostic patient profile

**Figure 2:**
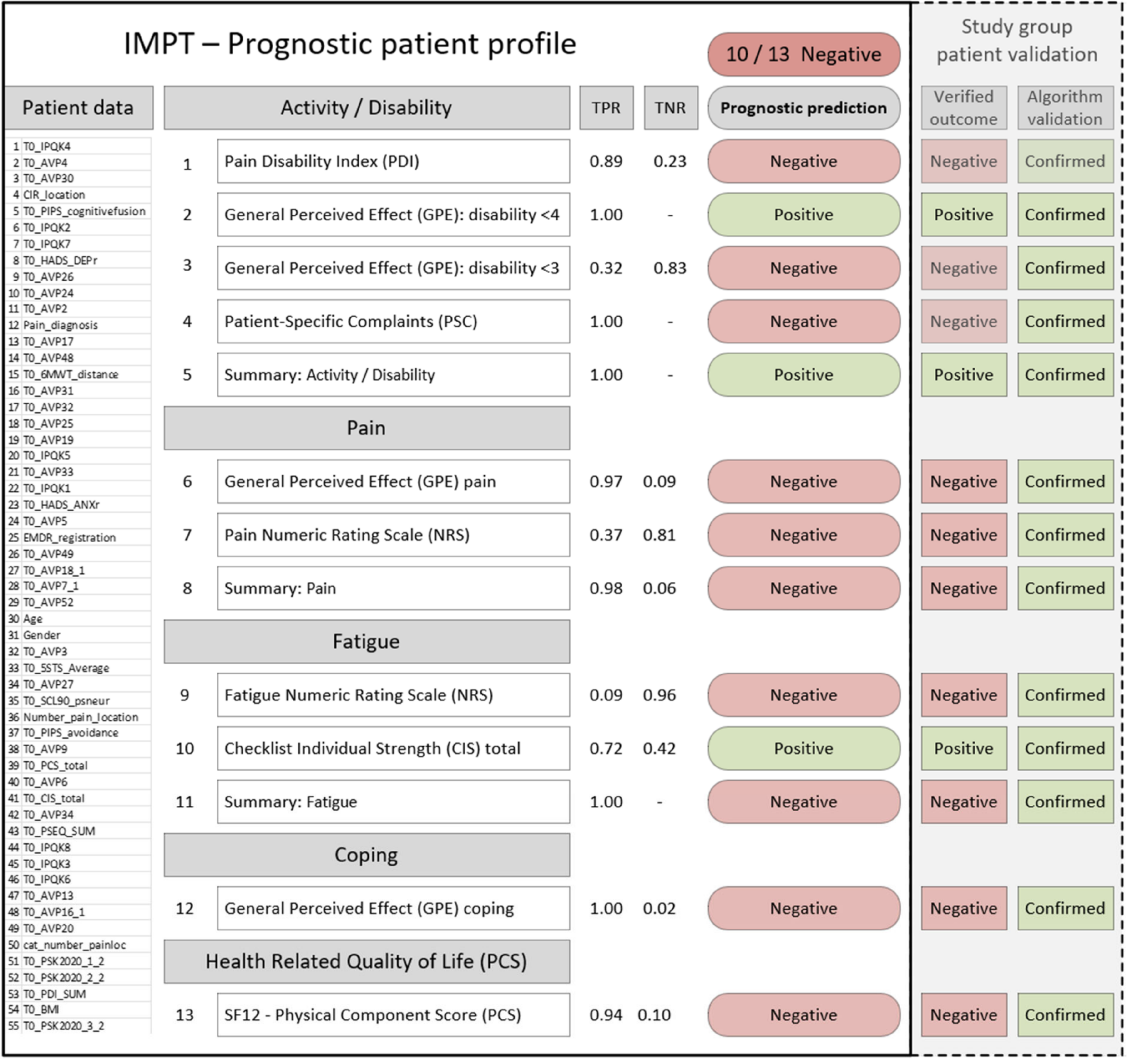
Example patient prognostic profile and validation with actual study data results. PDI, pain disability index; GPE, global perceived effect (disability); PSC, patient-specific complaints; NRS, numeric rating scale; CIS, checklist individual strength; SF-12 PCS, short-form quality of life survey physical component score. PDI, PSC, pain, fatigue, and CIS improvement are indicated by lower scores. GPE <3 indicates totally or much improved, GPE <4 indicates totally, much, or somewhat improved. TPR, true positive rate; TNR, true negative rate; patient data section is shown for illustrative purposes representing the 55 baseline prognostic variables and values that would appear on the patient profile.

The final machine learning framework provides the full set of 13 algorithms for each patient, establishing a stratified profile across outcome dimensions, see Figure 2. This includes estimated accuracy figures, true positive and negative rates for each algorithm, and the summary outcome count indicator. The profile also reflects the common situation in which patient assessment is not clear, as for most CIR program patients. The prognostic algorithm outcomes could be presented to assist clinicians with patient assessment, along with demographic and baseline prognostic variables. The left-hand section (solid black border) shows content available at patient assessment, with baseline variables, the predictive positive or negative outcomes for each measure, and the summary majority positive or negative scores. In this example, the right-hand section illustrates one of the clinician-reviewed patients with the study dataset outcomes and whether each algorithm was validated with a correct prediction. For this patient, the majority of algorithms (10/13) indicated negative outcomes. Predictions for each of the 13 algorithms were correctly confirmed with outcome data (Figure 2, dashed right-hand section), and individual clinician case file review for this patient verified that there had been no improvement from IMPT in this case.

The prognostic patient profile may assist clinicians in a few ways. In some cases, this could support assessment where the outcome is clearly positive or negative across dimensions. For mixed positive and negative outcome profiles, this subgroup of patients might benefit from further, more detailed assessment in particular dimensions or discussion of setting more specific program goals in target outcome measures. It is also a consideration that patients with a negative majority or mixed profile could form a supplementary assessment phase of the program where needed, for example, to undertake an initial pilot support of, say four weeks, for an interim review of progress before approval to proceed in the full 10-week program.

## 4. Discussion

We developed our pilot machine learning framework knowing that outcomes for programs supporting patients with CMP are highly variable and no single measure exists to consistently classify positive results. Clinicians assessing and developing individual patient support programs for this complex cohort consider multiple baseline dimensions and detailed patient backgrounds. There is no single reliable clinical measure to assess IMPT patient outcomes or therefore to provide an individual prognostic basis to identify patients most likely (or unlikely) to benefit from IMPT. In this context, our research was motivated by whether machine learning methods across several outcome measures could be combined to develop a stratified prognostic patient profile as a clinical decision support tool. The aim was to investigate whether collective algorithm models could, in some way, replicate the complex multidimensional assessment process clinicians undertake. In summary, we defined and developed our framework of 13 outcome measures and derived study group outcomes for each metric. These were then used to individually train each algorithm using the best 30 of 55 independently selected prognostic variables for each endpoint. We have reported and summarized prominent variables across all models and calculated estimated algorithm accuracy and error metrics using established random fivefold partitioning of the data. To verify results, we have developed a complete process of cross validation that retrospectively implemented each of our 13 algorithms on the deidentified source study group data by extracting only the prognostic variables that would have been available for baseline assessment.

As expected, the results of each of our 13 clinical endpoints and composite metrics confirmed that no individual outcome measure provided a consistently significant basis to assess newly referred patients. The individual experience of pain, fatigue, or restricted mobility is complex, and patient-reported program outcomes are perhaps subjective. Clinicians report patients completing the CIR program who are highly positive and feel they benefited from the program, but they often report mixed outcome measures across follow-up questionnaire domains. For this reason, the initial phase of the study was to define our ten clinical endpoints across the five relevant domains, as well as the three composite metrics. This was the starting point for assessing outcomes in the patient study dataset and develop target measures for supervised training of the algorithms.

Machine learning methods have been rapidly established in medical research with wide applications where clear clinical endpoints are identified, and large patient datasets are available to train algorithms. Applications of these methods have not surprisingly focused on areas where machine learning has clear strengths such as complex pattern recognition of well-labeled data. These types of machine learning applications have been used in chronic pain research, examining, for example, diagnostic imaging and assessment of patients suffering pain likely to progress to long-term chronic conditions (15, 33–35). Our machine learning study is novel as our CIR study group has already been diagnosed with CMP, and our patient cohort does not have a clear single outcome measure from IMPT participation. Our primary study question was whether machine learning could contribute to clinician assessment and patient discussions by establishing a prognostic stratified patient profile.

Our study aimed to leverage baseline patient questionnaires that are collected prior to patient assessment through an online portal. If algorithm-based predictors are of value, they could be made available in preparation for IMPT screening assessment as a prognostic patient profile. This would provide the basis for IMPT team discussions between the patient, psychologist, physiotherapist, and clinician (physiatrist) involved in the assessment process and decision of whether or not to start treatment. This could potentially assist clinicians and patients with individualized planning of goals through further utilizing detailed baseline data already available.

### 4.1. Complexity of measuring IMPT outcomes

Our research was motivated by the positive outcomes reported by many patients of the CIR program. As this chronic pain cohort has exhausted all available interventions, often over several years, it is significant to have any individual patient report clinically meaningful improvements from the 10-week program. The high proportion of patients completing the program reporting one or more positive outcomes, over 50% and as high as 85% for some outcome measures, is remarkable (3). However, IMPT outcomes are highly variable across multiple patient-reported dimensions, which clinicians assess to establish a baseline patient profile. In this context, our machine learning approach aimed to mimic a multidimensional approach to develop the collective set of stratified prognostic indicators to assist clinicians to assess new patient IMPT referrals.

Outcome heterogeneity is not a weakness in IMPT measurement; it reflects the complexity of clinical practice when assessing these patients with chronic pain. From our defined framework of 13 outcome measures, many patients achieve a majority of positive or negative indicators, while most report a mix. For this reason, our prognostic profile was not developed as a prescriptive clinical decision tool focused on a single metric. The framework of outcomes and thresholds for minimal clinically important change was based on clinical practice and published literature, as presented in Table 1 and Supplementary Table 1. Ongoing research is investigating refinement of IMPT outcome measures; for example, current practice defines a nine-point change in PDI as clinically meaningful, but nonlinear thresholds dependent on baseline score may provide improved accuracy across patients. Research investigating minimal clinically important change based on PDI baseline thresholds is in progress.

### 4.2. Improving IMPT patient outcomes

Having defined our framework of 13 IMPT patient outcome measures, the focus was to investigate whether machine learning could assist clinicians in improving patient outcomes further. Our study has emphasized the patient-focused nature of IMPT outcomes and the fundamental importance of active patient involvement in program planning. Patient engagement in the program approach, commitment to potential challenges, and the effort needed to change established beliefs and behaviors are critical to collaboratively designing individualized goals and building the skills and ability to learn to live with their chronic pain. The patient-focused perspective is fundamental to IMPT and similarly central to the design of the prognostic profile and how it might benefit patient pathways and outcomes. Our approach was not to develop a prescriptive algorithm-based metric that would categorically indicate whether a patient was suitable for IMPT or not. As we have presented, patient outcome heterogeneity is characteristic of IMPT programs and clinician assessment is complex and multifaceted. The implicit study question was whether machine learning could replicate a prognostic multidimensional patient profile across our 13 outcome measures.

While the prognostic profile does not aim to produce a composite indicator to suggest IMPT inclusion or exclusion, many patient results produced a consistent positive or negative outcome majority. This provides the overarching perspective of the profile and possible pathways of how patient outcomes might be improved. If the majority of prognostic outcomes are positive, the patient assessment direction is encouraging, and, in many cases, it is expected to be consistent with a preliminary review. In this scenario, the prognostic profile could help verify preliminary clinician assessment and provide the stratified outcome measures to help facilitate clinician and patient discussion of specific program goals. Our example patient profile illustrates the prognostic prediction for each of our 13 outcomes, grouped across the five measurement domains, as presented in Figure 2. This example shows that most predicted outcomes are negative, suggesting that this patient may be unlikely to benefit from the IMPT program. As part of our algorithm validation process, we intentionally chose to examine this challenging subgroup where most indicators were negative and where each algorithm was correctly validated against actual patient outcomes. From this targeted subgroup of 81 negative profiles, a separate clinician case file review confirmed that half of the patients did not achieve a clinically significant improvement, with the remaining half reporting mixed outcomes for further review. Our patient profile example was included in the clinician case file review and confirmed that this patient did not go on to achieve a clinically meaningful improvement. The open question is whether this result could have been different if the prognostic profile was available at baseline assessment to feed into goal-setting and discussion of realistic expectations of program success.

The prognostic measures also present estimated accuracy for each outcome, for clinician consideration, given that some algorithms were trained on high proportions of positive endpoints and therefore reflected equally high true positive or true negative rates. This is not an algorithm weakness but an expected replication of commonly observed patient characteristics. The accuracy metrics are provided for transparency and perhaps mirror clinician considerations where very likely attributes are discounted in the overall assessment profile. We have reported all measures investigated in our study, irrespective of individual algorithm accuracy. Further research could refine the profile summary, perhaps by developing supplementary composite indicators weighted by algorithm accuracy.

### 4.3. Helping develop treatment plans

Detailed baseline assessment of the prognostic patient profile is anticipated to better support clinician and patient collaborative discussion, planning, and goal-setting. In our patient example, where most prognostic algorithms indicated a negative outcome, this is not necessarily intended to discourage a patient from entering the program. Excluding patients is not the objective; improving patient outcomes for all referred patients is the primary goal. This said, if a prognostic profile of consistently negative outcomes is discussed with a patient, they may feel that they do not wish to proceed with the IMPT path of treatment. The program requires substantial commitment over 10 weeks, so patients need to be comfortable with their expected investment in time and related prospects of success. Further, in cases indicating negative profiles, clinicians observe that some patients struggle with IMPT and can become despondent when they fail to make progress and may progressively feel discouraged. Helping reduce IMPT intake for some patients who are unlikely to benefit remains an important consideration but would be decided, as always, through clinician and patient discussion. Based on our study group, the proportion of patients with a consistently negative leaning profile is relatively small, at around 9% of new patient assessments.

The assessment and patient discussion may be more nuanced in the case of mixed predictive profiles of positive and negative outcomes. For example, clinicians report that some patients request treatment through the IMPT program, even when baseline assessment suggests that the likelihood of positive results is low. Some patients are aware of IMPT positive outcomes and have genuine intent to pursue similar positive progress for their chronic pain. These patients could review the prognostic profile with the clinician and discuss the reasons why the program might not be effective for them. This potentially feeds back into patient engagement, where realistic targets could be considered. Perhaps negative prognostic profiles will motivate some patients to reassess IMPT commitment and actively encourage the process of setting (and then achieving) program goals. A further consideration might be introducing an interim pilot IMPT phase of, say 4 weeks, to allow patients to demonstrate their motivation to take responsibility and achieve their goals. Interim results could then be reviewed with the clinician at the end of the 4 weeks to reassess and agree about proceeding into the complete standard 10-week program.

The prognostic profiles could also assist clinicians across program locations, areas of clinical focus, and ongoing development or refinement of multidimensional outcome measures. The framework provides the foundation for the reiterative development of new prognostic factors and outcome measures, potentially used to retrain algorithms as additional endpoints. The prognostic framework of measures may also assist with the consistency of standardized baseline assessment. Also, periodically, new or improved measures could flow through to IMPT teams, especially if the program is being expanded to new locations and new centers. Our current prognostic framework is the starting point, and it is envisaged to continue to develop with ongoing outcome measurement research. The addition of new or refined clinical endpoints is separate from ongoing reinforced machine learning that can be retrained periodically as further patient data becomes available from new patients and new treatment centers to further validate and refine algorithms.

### 4.4. IMPT prognostic factors

We took an open approach to selecting prognostic variables to train each algorithm. In addition to including core data items based on clinician advise, we also expanded the prognostic factor pool by including all variables available in the study group dataset considered to have potential predictive value. We took this wider approach as the full set of 55 candidate variables was subject to subsequent minimum redundancy maximum relevance feature selection. This process reduced the number of features to 30 variables with the highest prognostic value for each algorithm, so prognostic factors varied across each model. Our set of machine learning prognostic variables included data items reported in current evidence of regression-based IMPT prognostic factors (36, 37). Summarizing across all 13 algorithms resulted in three baseline prognostic variables relevant for all models and five variables relevant for 92% of models. Other prognostic factors were selected in more than 75% of models. Age and gender were selected in around half of the trained models, while patient BMI was not selected in any. These results are partly in line with a recent meta-analysis of prognostic factors for IMPT, a Swedish cohort study based on conventional regression methods examining improved functioning following interdisciplinary rehabilitation (36, 37), and regression-based prognostic variables for chronic low back pain conservative treatment indicating two pretreatment significant predictive factors of employment status and having mild to moderate disability (38, 39).

Remarkably, some variables were previously not identified, such as CIR location, which means that despite a protocolized treatment that can be individualized, outcome success is dependent on the location where a patient is treated. This calls for further analysis. Furthermore, the worst pain intensity and cognitive fusion do seem to matter, which might be due to the fact that a big part of the CIR treatment uses Acceptance and Commitment Therapy, which helps the patient to defuse the pain and accept it. Also, very interesting is the influence of age, gender, and duration of pain, which previously have not been identified. Further, pain catastrophizing, one of the most often mentioned prognostic factors, was only relevant in four of the 13 models. The mix of prognostic variables suggests that machine learning feature selection may help reduce or offset the bias of imbalanced baseline characteristics. The algorithms focus on prognostic significance for each outcome, so the high proportion of female or overweight patients, for example, did not result in corresponding high prognostic importance. This is also interesting as it indicates that machine learning-based research can help overcome some potential bias. For example, clinicians have observed medical advisor reluctance to offer IMPT to patients with chronic pain who have a BMI over 35. Our results clearly show that excluding patients from IMPT based on bodyweight is not supported by evidence, consistent with our machine learning prognostic profiles, which are not influenced by BMI.

### 4.5. Improving program outcomes and efficiency

As the program provides 10 weeks of individualized intensive support, it is a high-cost intervention, and the interdisciplinary teams take time to develop and scale to increase capacity and geographical reach. A supplementary study question was whether our machine learning prognostic patient profile could potentially assist clinicians in further optimizing current capacity through supporting patient selection and individual goal-setting. Well-established health economic evaluation methods help assess efficiency by comparing intervention costs with program effectiveness in improving patient health outcomes. Preliminary research indicates the CIR program is likely cost-effective (2), and related types of chronic pain interventions have also been reported to be cost-effective (40).

Evaluating the cost-effectiveness of the IMPT program reflects the complexity of the intervention, variation across multiple outcome measures, and the related heterogeneity of patient outcomes. Despite this, further improvement in a small proportion of patients’ selection and goal-setting could potentially contribute to the effectiveness and related cost-effectiveness of the IMPT program. Health economic methods are being developed to examine personalized interventions such as the individualized IMPT program (41–43). This presents a further potential direction for assessing stratified heterogeneous outcomes, perhaps providing more targeted subgroups for ongoing machine learning endpoints and refined algorithms in the prognostic patient profile. This work could potentially help illuminate especially effective (and cost-effective) subgroups otherwise masked when evaluating the program across total study group average treatment effects.

Improved IMPT outcomes, including a faster return to work, have been reported through multidisciplinary interventions compared to brief interventions (44) and increased work days in patients with nonacute nonspecific low back pain (45). There is anecdotal evidence in the patient study group of successfully returning to work following positive outcomes from the IMPT program. Given the average patient age of 44 years, many have the prospect of decades of productive working life ahead. Although employment pathways are subject to longitudinal follow-up, preliminary observation immediately following IMPT completion indicates that around 200 patients from the study group had fully returned to work (*n* = 2,364; ∼8%). Furthermore, 50.3% reported a clinically meaningful increase in self-rated work capacity up to 1 year post-treatment. Current longitudinal follow-up of CIR patients is assessing these self-rated work capacity results and indicates the improved outcomes are mostly sustained at 12 months postprogram (6). If this return-to-work proportion is validated and potentially scaled to countrywide coverage in the Netherlands, the IMPT program could potentially increase around fourfold, suggesting a possible return to career pathways for several hundred patients. Program longitudinal follow-up also verifies the sustained positive improvements across all outcome measures including health-related quality of life (6). These types of program results represent potentially significant benefits from a societal perspective. Ongoing health economic evaluation could further investigate the upfront IMPT investment in the context of these types of system-wide benefits, which are often diffused, difficult to measure in financial terms, and may be sustained over future years. An overarching societal health economic perspective is important as IMPT funding in the Netherlands is provided through healthcare insurance entities, whereas substantial ongoing positive outcomes may result in the wider healthcare system, government support agencies, employment sectors, taxation collection, and other quality of life and social benefits.

### 4.6. Clinical decision support for IMPT

Alongside developing our 13 outcome measures and prognostic patient profile, we have considered throughout the project how this type of machine learning decision support could be implemented to assist clinicians and patients. Despite the accelerating pace of development in machine learning methods in healthcare, introduction into clinical practice remains challenging due to the “blackbox” perception and the need to carefully validate results prior to implementation (16, 46). With this in mind, we considered potential implementation through close clinician collaboration across all phases of the project. This has included clinician guidance and discussion at all stages of development including approach and definition of outcome measures, algorithm model features, multiple layers of result validation, and frequent discussion on how our machine learning prognostic patient profile might assist clinicians and patients in practice. It seems intuitive but recent research emphasizes that clinician expert involvement in design and development phases of machine learning-based predictive support systems should be explicitly reported to establish the integrity of results and help overcome implementation challenges (47).

The blackbox impression of machine learning is often cited as an obstacle to implementation with the argument it is not acceptable to base clinical decisions on mechanisms where the causal pathway is not clearly understood. Core machine learning methods are well established, and the theory of classification algorithms used in this study is well documented as for alternative advanced statistical and regression model methods (48). Medical practice routinely adopts clinical trial evidence based on sophisticated statistics, and clinicians are not expected to prove causal pathways of all methods. The same applies to commonly undertaken medical interventions; for example, in pain management, the exact molecular mechanisms behind general anesthetic drugs are not well understood but widely used as the effectiveness, risks, and dosage control are well established in clinical practice (49). In a similar perspective, we developed an additional layer of validation based on a comparison of our prognostic algorithms with source patient outcome data to produce a subgroup sample of results for clinician review. Irrespective of proving how algorithms function, we wanted an independent practical test of whether they contribute to prognostic patient assessment. We selected the most challenging subgroup of 81 patients with mostly negative prognostic measures as they had already received different noneffective treatments and were identified as complex (WPN3 and WPN4, meaning that psychological and social factors are contributing to moderate to high levels of their pain and disability). This group is also challenging because our outcome measures, as defined, achieved high proportions of positive outcomes for one or more of the clinical endpoints. This imbalance in training data results in corresponding high levels of true positive predictions making negative outcomes more difficult to identify. As the profile includes some measures where outcomes are more balanced or predominantly negative, it appears that the collective profile is still able to make a prognostic contribution. This independent validation confirmed that half of the 81 patients had not achieved a positive change and the remaining half reported mixed results for further assessment. This is a significant result as correctly predicting any patient in this complex subgroup at baseline assessment would be considered positive, and identification of half the group is exceptional. Correctly validating this substantial proportion of our intentionally targeted negative outcome sample was an intriguing finding. This highlights the potential prognostic profile value if it had been available for these patients during baseline assessment, as it was confirmed they did not go on to achieve positive outcomes from the program.

Continuing on the goal of potential clinical implementation and establishing confidence in the prognostic profile, we considered a recently developed a clinician checklist to help assess the suitability of machine learning applications in healthcare (18). This research provides 10 questions clinicians can ask without having to be experts in machine learning methods including the purpose of the algorithms, data quality and quantity, algorithm performance and transferability to new settings, clinical intelligibility of outcomes, how they might fit with patient workflow and improving outcomes, risks of patient harm, and ethical, legal, and social concerns. The purpose of our algorithms is to provide our prognostic patient profile at baseline assessment. The patient dataset has been developed and maintained by the CIR that manage the IMPT program giving confidence in data quality and providing a large sample of patients from 2019 to 2021 (*n* = 2,364). We have presented algorithm performance measures with each algorithm for clinician reference across the profile. Transferability of algorithms to a new setting is not relevant as we have individually trained all 13 algorithms for use in our prognostic IMPT profile. Results are presented in the stratified profile across outcome dimensions to assist intelligibility, with a binary positive or negative indicator based on a predicted clinically significant outcome for each measure, as shown in Figure 2. As discussed above, we have considered how the prognostic profile could be used in practice to improve patient outcomes. As the patient profile is not presented as a prescriptive decision tool, patient care decisions are always with the clinician, so the patient risk from the algorithms is not a material concern. Also, IMPT is not a high-risk intervention but a personalized support program where all other clinical options have been exhausted. In line with low patient risk from the profile algorithms, and patient decisions remaining with the clinician and patient, there are no significant ethical, legal, or social concerns with the use of the prognostic profile (50).

Separately, we examined machine learning implementation issues including transparency of algorithms, and biases in supervised methods for machine learning in chronic pain research (15, 51). There are recognized concerns that machine learning methods may conceal how algorithms are influenced through bias in training data sources (52). For our project, we are not using large linked administrative data, and as our patient study dataset is collected and managed by CIR, we are confident the source training data are accurate. We have considered the respective advantages of machine learning in our context including strengths in diagnosis, classification, and related predictions, as well as challenges with data preprocessing, algorithm model training and refinement with respect to the actual clinical problem (53), and how machine learning may influence clinician treatment selections (54). To our knowledge, this study is the first research applying machine learning to prognostically assess IMPT patient outcomes at baseline and through our multidimensional framework of 13 measures.

Overall, although no single outcome measure provided a reliable indicator, the collective 13 algorithms established a stratified, prognostic patient profile across the five clinically relevant domains. Even though no single algorithm was assessed as having high levels of accuracy, patient-level validation showed that the collective profile achieved consistent prognostic assessment of original source outcomes for three-quarters (75.3%) of the study group.

We see multiple potential benefits from predictive indicators as a clinician decision support tool during baseline patient assessment. Our study has presented examples of how the prognostic patient profile could support assessment where the outcome is clearly positive or negative across outcome dimensions and mixed positive and negative outcome profiles. In summary, we have considered throughout the project how this prognostic profile might be implemented as a clinical decision support tool to assist with IMPT patient assessment and goal-setting and potentially contribute to further improvement in patient outcomes.

### 4.7. Limitations

The most prominent characteristic of the study is the inherent heterogeneity and complexity in IMPT patient outcomes. The intricacy of CMP results in substantial variation across outcomes, and there is no established consensus in some measures. We developed 10 clinical endpoints in the patient dataset based on clinical practice and clinician expert advice. Some patients report improvements in a specific measure and not others, and the psychometric associations are not clearly understood.

The prognostic machine learning models reflect the complex clinician assessment process and consideration of patient-specific program management pathways. This is not a design limitation, and we have intentionally targeted this complex study group to investigate potential clinical decision support through our multidimensional framework of algorithms.

The defined clinical endpoints resulted in a high proportion of positive results for many outcome measures. This produced imbalanced training patient datasets, impacting the capacity of the algorithms to distinguish between positive and negative outcomes. Furthermore, the current project is based on patient-reported outcome measures, which are often complex and subjective. For these reasons, the endpoint framework was developed to provide sufficient multiple dimensions and reduce dependence on some individual less accurate models.

Machine learning classification models are pattern recognition models based on response variables without the inference of causality. Separately, we used all potential prognostic variables available in the patient dataset, and not only based on current evidence of regression-based prognostic factors. We believe that this approach is more likely a strength than a limitation, but we recognize that including a high number of prognostic variables in machine learning can lead to overfitting of models and potentially reduced predictive performance of algorithms when used with new patient data. We consider that our approach of reducing prognostic factors to the 30 best-performing variables per model through feature selection and fivefold cross validation in algorithm training has minimized overfitting but acknowledge as a potential limitation in machine learning.

The patient study group data include only patients who have been referred to and participated in the CIR program. A comparison group or data for patients referred to the program that did not proceed is not currently available. Finally, relating to the health economic perspective of IMPT, reliable employment data are not presently available for this study and require ongoing longitudinal follow-up to verify the return to work rates and whether employment outcomes are sustained in the long term.

### 4.8. Ongoing and future research directions

This is a preliminary machine learning study to develop and assess a pilot framework of predictive indicators for our prognostic patient profile. The project has established promising results and provides the foundation for ongoing future research in multiple directions. Ongoing longitudinal follow-up and collection of new patient data will further validate the accuracy of the pilot algorithm models. The ongoing data may be used for the refinement of clinical endpoint measures as well as source data for reinforced algorithm training, which may further improve predictive models over time as more data become available.

Potential refinement or development of additional outcome measures, for example, nonlinear PDI thresholds using baseline scores, will potentially provide the basis for retraining algorithms or providing new supplementary profile prognostic models. Potential additional machine learning models could examine IMPT time series data using more advanced neural network models. The prognostic algorithms could be developed as a standalone module or potentially integrated with baseline data available in the CIR IMPT online patient portal to provide a pilot decision support tool for clinicians when assessing new patients.

There are also multiple possible extensions for further health economic cost-effectiveness modeling, integrating potential patient outcome improvements, subgroup analysis of patient heterogeneity, and extended societal perspectives. This further health economic research could assess significant anecdotal benefits along medium- and longer-term pathways where patients resume higher function, achieve a return to employment or faster return to employment than otherwise, and have improved quality of life.

## Data Availability

The study group patient dataset is confidentially managed by CIR and is not available for public access. Requests to access the datasets should be directed to the corresponding author.

## References

[1] World Health Organization. Musculoskeletal health. Fact sheet (2022). Accessed at: https://www.who.int/news-room/fact-sheets/detail/musculoskeletal-conditions (Accessed April 22, 2023).

[2] Volker G van Vree F Wolterbeek R van Gestel M Smeets R Koeke A Long-term outcomes of multidisciplinary rehabilitation for chronic musculoskeletal pain. Musculoskeletal Care. (2017) 15(1):59–68. 10.1002/msc.1141 27098842

[3] Elbers S Wittink H Konings S Kaiser U Kleijnen J Pool J Longitudinal outcome evaluations of interdisciplinary multimodal pain treatment programmes for patients with chronic primary musculoskeletal pain: a systematic review and meta-analysis. Eur J Pain. (2022) 26(2):310–35. 10.1002/ejp.1875 34624159 PMC9297911

[4] Kamper SJ Apeldoorn AT Chiarotto A Smeets RJ Ostelo RW Guzman J Multidisciplinary biopsychosocial rehabilitation for chronic low back pain: Cochrane systematic review and meta-analysis. Br Med J. (2015) 350:h444. 10.1136/bmj.h444 25694111 PMC4353283

[5] Breugelmans L Scheffer E Beckers LWME Oosterwijk RFA Nijland G Smeets RJEM. Systematic description of an interdisciplinary multimodal pain treatment programme for patients with chronic musculoskeletal pain, using the TIDieR checklist. BMC Res Notes. (2022) 15(1):320. 10.1186/s13104-022-06211-z 36221116 PMC9551242

[6] Smeets RJEM Oosterwijk RFA. Lange termijn resultaten van een medisch specialistisch pijnrevalidatieprogramma. Nederlandstalig Tijdschrift Pijnbestrijding. (2021) 40(82):6–14.

[7] Koele R Volker G van Vree F van Gestel M Köke A Vliet Vlieland T. Multidisciplinary rehabilitation for chronic widespread musculoskeletal pain: results from daily practice. Musculoskeletal Care. (2014) 12(4):210–20. 10.1002/msc.1076 24916665

[8] Ringqvist Å Dragioti E Björk M Larsson B Gerdle B. Moderate and stable pain reductions as a result of interdisciplinary pain rehabilitation—a cohort study from the Swedish Quality Registry for Pain Rehabilitation (SQRP). J Clin Med. (2019) 8(6). 10.3390/jcm8060905 PMC6617026 31238588

[9] Preis MA Vögtle E Dreyer N Seel S Wagner R Hanshans K Long-term outcomes of a multimodal day-clinic treatment for chronic pain under the conditions of routine care. Pain Res Manag. (2018) 2018:9472104. 10.1155/2018/9472104 29808108 PMC5901829

[10] Selya A Anshutz D Griese E Weber TL Hsu B Ward C. Predicting unplanned medical visits among patients with diabetes: translation from machine learning to clinical implementation. BMC Med Inform Decis Mak. (2021) 21(1):111. 10.1186/s12911-021-01474-1 33789660 PMC8011134

[11] Aggarwal N. Prediction of low back pain using artificial intelligence modeling. J Med Artif Intell. (2021) 4. 10.21037/jmai-20-55

[12] Tagliaferri SD Angelova M Zhao X Owen PJ Miller CT Wilkin T Artificial intelligence to improve back pain outcomes and lessons learnt from clinical classification approaches: three systematic reviews. NPJ Digit Med. (2020) 3(1):93. 10.1038/s41746-020-0303-x 32665978 PMC7347608

[13] Oude Nijeweme-d'Hollosy W van Velsen L Poel M Groothuis-Oudshoorn CGM Soer R Hermens H. Evaluation of three machine learning models for self-referral decision support on low back pain in primary care. Int J Med Inform. (2018) 110:31–41. 10.1016/j.ijmedinf.2017.11.010 29331253

[14] Mork PJ Bach K. A decision support system to enhance self-management of low back pain: protocol for the selfBACK project. JMIR Res Protoc. (2018) 7(7):e167. 10.2196/resprot.9379 30030208 PMC6076372

[15] Jenssen MDK Bakkevoll PA Ngo PD Budrionis A Fagerlund AJ Tayefi M Machine learning in chronic pain research: a scoping review. Appl Sci. (2021) 11(7):3205. 10.3390/app11073205

[16] The Lancet. Artificial intelligence in health care: within touching distance. Lancet. (2017) 390(10114):2739. 10.1016/S0140-6736(17)31540-4 29303711

[17] Giordano C Brennan M Mohamed B Rashidi P Modave F Tighe P. Accessing artificial intelligence for clinical decision-making. Front Digit Health. (2021) 3. 10.3389/fdgth.2021.645232 34713115 PMC8521931

[18] Scott I Carter S Coiera E. Clinician checklist for assessing suitability of machine learning applications in healthcare. BMJ Health Care Inform. (2021) 28(1):e100251. 10.1136/bmjhci-2020-100251 33547086 PMC7871244

[19] Köke AJ Smeets RJ Schreurs KM van Baalen B de Haan P Remerie SC Dutch dataset pain rehabilitation in daily practice: content, patient characteristics and reference data. Eur J Pain. (2017) 21(3):434–44. 10.1002/ejp.937 27634023

[20] Pollard CA. The relationship of family environment to chronic pain disability. San Diego, CA: California School of Professional Psychology (1981).

[21] Soer R Reneman MF Vroomen PC Stegeman P Coppes MH. Responsiveness and minimal clinically important change of the pain disability index in patients with chronic back pain. Spine. (2012) 37(8):711–5. 10.1097/BRS.0b013e31822c8a7a 21796022

[22] Copay AG Subach BR Glassman SD Polly DW Schuler TC Jr. Understanding the minimum clinically important difference: a review of concepts and methods. Spine J. (2007) 7(5):541–6. 10.1016/j.spinee.2007.01.008 17448732

[23] Beurskens A. A patient-specific approach for measuring functional status in low back pain. Low back pain and traction. Thesis. Maastricht: Rijksuniversiteit Limburg (1996). p. 83–96.

[24] Beurskens AJ de Vet HC Köke AJ Lindeman E van der Heijden GJ Regtop W A patient-specific approach for measuring functional status in low back pain. J Manipulative Physiol Ther. (1999) 22(3):144–8. 10.1016/S0161-4754(99)70127-2 10220712

[25] Cohen J. Statistical power analysis for the behavioral sciences. New York: Routledge (2013).

[26] Ostelo RW Deyo RA Stratford P Waddell G Croft P Von Korff M Interpreting change scores for pain and functional status in low back pain: towards international consensus regarding minimal important change. Spine. (2008) 33(1):90–4. 10.1097/BRS.0b013e31815e3a10 18165753

[27] Vercoulen JH Swanink CM Fennis JF Galama JM van der Meer JW Bleijenberg G. Dimensional assessment of chronic fatigue syndrome. J Psychosom Res. (1994) 38(5):383–92. 10.1016/0022-3999(94)90099-X 7965927

[28] Ware J Jr. Kosinski M Keller SD. A 12-item short-form health survey: construction of scales and preliminary tests of reliability and validity. Med Care. (1996) 34(3):220–33. 10.1097/00005650-199603000-00003 8628042

[29] Chen R-C Dewi C Huang S-W Caraka RE. Selecting critical features for data classification based on machine learning methods. J Big Data. (2020) 7(1):52. 10.1186/s40537-020-00327-4

[30] Ding C Peng H. Minimum redundancy feature selection from microarray gene expression data. J Bioinform Comput Biol. (2005) 3(2):185–205. 10.1142/S0219720005001004 15852500

[31] Shengping Y Gilbert B. The receiver operating characteristic (ROC) curve. Southwest Respir Crit Care Chron. (2017) 5(19):34–6. 10.12746/swrccc.v5i19.391

[32] Nederlandse vereniging van revalidatieartsen. Behandelkader Chronische pijn zich uitend in het houdings- en bewegingsapparaat (2020). Available at: https://www.revalidatie.nl/wp-content/uploads/2022/10/behandelkader_chronische_pijn_versie_28-9-2020_-_goedgekeurd_in_alv_19-11-2020.pdf (Accessed April 21, 2023).

[33] Shim J-G Ryu K-H Cho E-A Ahn JH Kim HK Lee Y-J Machine learning approaches to predict chronic lower back pain in people aged over 50 years. Medicina. (2021) 57(11):1230. 10.3390/medicina57111230 34833448 PMC8618953

[34] D'Antoni F Russo F Ambrosio L Vollero L Vadalà G Merone M Artificial intelligence and computer vision in low back pain: a systematic review. Int J Environ Res Public Health. (2021) 18(20):10909. 10.3390/ijerph182010909 34682647 PMC8535895

[35] Rajkomar A Dean J Kohane I. Machine learning in medicine. N Engl J Med. (2019) 380(14):1347–58. 10.1056/NEJMra1814259 30943338

[36] Tseli E Boersma K Stålnacke BM Enthoven P Gerdle B Äng BO Prognostic factors for physical functioning after multidisciplinary rehabilitation in patients with chronic musculoskeletal pain: a systematic review and meta-analysis. Clin J Pain. (2019) 35(2):148–73. 10.1097/AJP.0000000000000669 30371517 PMC6343958

[37] Tseli E Vixner L LoMartire R Grooten WJA Gerdle B Äng BO. Prognostic factors for improved physical and emotional functioning one year after interdisciplinary rehabilitation in patients with chronic pain: results from a national quality registry in Sweden. J Rehabil Med. (2020) 52(2):jrm00019. 10.2340/16501977-2648 31995224

[38] van Hooff ML Spruit M O'Dowd JK van Lankveld W Fairbank JC van Limbeek J. Predictive factors for successful clinical outcome 1 year after an intensive combined physical and psychological programme for chronic low back pain. Eur Spine J. (2014) 23(1):102–12. 10.1007/s00586-013-2844-z 23771553 PMC3897840

[39] van Hooff ML van Loon J van Limbeek J de Kleuver M. The Nijmegen decision tool for chronic low back pain. Development of a clinical decision tool for secondary or tertiary spine care specialists. PLoS One. (2014) 9(8):e104226. 10.1371/journal.pone.0104226 25133645 PMC4136789

[40] Hochheim M Ramm P Wunderlich M Amelung V. Cost-effectiveness analysis of a chronic back pain multidisciplinary biopsychosocial rehabilitation (MBR) compared to standard care for privately insured in Germany. BMC Health Serv Res. (2021) 21(1):1362. 10.1186/s12913-021-07337-9 34952585 PMC8705190

[41] Kohli-Lynch CN Briggs AH. Heterogeneity in cost-effectiveness analysis. Oxford, UK: Oxford University Press (2019).

[42] Grutters JPC Sculpher M Briggs AH Severens JL Candel MJ Stahl JE Acknowledging patient heterogeneity in economic evaluation: a systematic literature review. PharmacoEconomics. (2013) 31(2):111–23. 10.1007/s40273-012-0015-4 23329430

[43] Bonander C Svensson M. Using causal forests to assess heterogeneity in cost-effectiveness analysis. Health Econ. (2021) 30(8):1818–32. 10.1002/hec.4263 33942950

[44] Brendbekken R Eriksen HR Grasdal A Harris A Hagen EM Tangen T. Return to work in patients with chronic musculoskeletal pain: multidisciplinary intervention versus brief intervention: a randomized clinical trial. J Occup Rehabil. (2017) 27(1):82–91. 10.1007/s10926-016-9634-5 26910406 PMC5306180

[45] Kool J Bachmann S Oesch P Knuesel O Ambergen T de Bie R Function-centered rehabilitation increases work days in patients with nonacute nonspecific low back pain: 1-year results from a randomized controlled trial. Arch Phys Med Rehabil. (2007) 88(9):1089–94. 10.1016/j.apmr.2007.05.022 17826451

[46] Ghassemi M Oakden-Rayner L Beam AL. The false hope of current approaches to explainable artificial intelligence in health care. Lancet. (2021) 3(11):e745. 10.1016/S2589-7500(21)00208-9 34711379

[47] Schwartz JM Moy AJ Rossetti SC Elhadad N Cato KD. Clinician involvement in research on machine learning-based predictive clinical decision support for the hospital setting: a scoping review. J Am Med Inform Assoc. (2021) 28(3):653–63. 10.1093/jamia/ocaa296 33325504 PMC7936403

[48] Flach P. Machine learning: the art and science of algorithms that make sense of data. Cambridge: Cambridge University Press (2012).

[49] Pavel MA Petersen EN Wang H Lerner RA Hansen SB. Studies on the mechanism of general anesthesia. Proc Natl Acad Sci U S A. (2020) 117(24):13757–66. 10.1073/pnas.2004259117 32467161 PMC7306821

[50] Vayena E Blasimme A Cohen IG. Machine learning in medicine: addressing ethical challenges. PLoS Med. (2018) 15(11):e1002689. 10.1371/journal.pmed.1002689 30399149 PMC6219763

[51] Faes L Liu X Wagner SK Fu DJ Balaskas K Sim DA A clinician's guide to artificial intelligence: how to critically appraise machine learning studies. Transl Vis Sci Technol. (2020) 9(2):7. 10.1167/tvst.9.2.7 32704413 PMC7346877

[52] Gianfrancesco MA Tamang S Yazdany J Schmajuk G. Potential biases in machine learning algorithms using electronic health record data. JAMA Intern Med. (2018) 178(11):1544–7. 10.1001/jamainternmed.2018.3763 30128552 PMC6347576

[53] Ngiam KY Khor IW. Big data and machine learning algorithms for health-care delivery. Lancet Oncol. (2019) 20(5):e262–e73. 10.1016/S1470-2045(19)30149-4 31044724

[54] Jacobs M Pradier MF McCoy TH Jr. Perlis RH Doshi-Velez F Gajos KZ. How machine-learning recommendations influence clinician treatment selections: the example of the antidepressant selection. Transl Psychiatry. (2021) 11(1):108. 10.1038/s41398-021-01224-x 33542191 PMC7862671

