# Corrigendum: Machine learning clinical decision support for interdisciplinary multimodal chronic musculoskeletal pain treatment

**DOI:** 10.3389/fpain.2023.1327997

**Published:** 2023-11-27

**Authors:** Fredrick Zmudzki, Rob J. E. M. Smeets

**Affiliations:** ^1^ Époque Consulting, Sydney, NSW, Australia; ^2^ Social Policy Research Centre, University of New South Wales, Sydney, NSW, Australia; ^3^ Department of Rehabilitation Medicine, Care and Public Health Research Institute (CAPHRI), Faculty of Health, Life Sciences and Medicine, Maastricht University, Maastricht, Netherlands; ^4^ CIR Rehabilitation, Eindhoven, Netherlands; ^5^ Pain in Motion International Research Group (PiM), Brussels, Belgium

**Keywords:** chronic pain, musculoskeletal pain, machine learning, interdisciplinary care, clinical decision support, prognosis, outcome

A Corrigendum on Machine learning clinical decision support for interdisciplinary multimodal chronic musculoskeletal pain treatment By Zmudzki F and Smeets RJEM (2023) Machine learning clinical decision support for interdisciplinary multimodal chronic musculoskeletal pain treatment. Front. Pain Res. 4:1177070. doi: 10.3389/fpain.2023.1177070

In the published article, there was a mistake in the corresponding author email address for author Rob J. E. M. Smeets. The email was incorrectly displayed as “r.smeets@maastrtichtuniversity.nl” The correct email address is: “r.smeets@maastrichtuniversity.nl”

The authors apologize for this error and state that this does not change the scientific conclusions of the article in any way. The original article has been updated.

