# Supplementary material for: Machine learning clinical decision support for interdisciplinary multimodal chronic musculoskeletal pain treatment
Source: Front Pain Res (Lausanne). 2023 May 9;4:1177070. doi: 10.3389/fpain.2023.1177070 (PMC10203229; doi:10.3389/fpain.2023.1177070)
Supplement: Supplementary file 1 [file Table1.docx]

| **Measure Number** | **Measurement instrument** | **Measurement domain** | **Measurement construct** | **Response scale** | **Score** | **Metric definition** |
| --- | --- | --- | --- | --- | --- | --- |
| 1.1 | PDI (18) | *Disability* | Measures to what degree pain prevents the patient from participating in daily activities | 0-10 scale for seven categories of activities | A sum score is calculated, ranging from 0 to 70. Lower scores mean lower disability | A decrease of 9 or more points (19) |
| 1.2 | GPE Disability <4 | *Disability* | Measures to what degree the patient reports improvement compared to the start of the treatment with regard to limitations in daily functioning due to the pain | 1-7 scale | Lower scores mean more improvement | Patients with scores 1, 2 or 3 are considered responders (20) |
| 1.2a | GPE Disability <3 | *Disability* | Same as 1.2 above | 1-7 scale | Lower scores mean more improvement | Patients with scores 1 or 2 are considered responders (20) |
| 1.3 | PSC (21, 22) | *Disability / Activity* | Measures a patient’s functional status by asking, for three self-selected daily activities, to what degree the patient is limited in these activities by his/her pain complaints.  Limitation per activity is scored on a 0-100 visual analogue scale (VAS) | Lower scores mean fewer limitations | The average of three VAS-scores is calculated | Effect size Cohen > 0.8 (23) |
| C1 | Measure 1.1 or 1.2 or 1.3 | *As above* | As above for each measure | As above | A positive outcome if any one of the measures is positive | As above |
| 2.1 | GPE Pain | *Pain* | Measures to what degree the patient reports improvement compared to the start of the treatment with regard to pain | 1-7 global perceived effect scale | Lower scores mean more improvement | Patients with scores 1, 2 or 3 are considered responders (20) |
| 2.2 | NRS Pain last week | *Pain* | Records the average level of pain during the last week | 0-10 numerical rating scale | Lower scores mean less pain | Reduction of 30% compared to baseline (24) |
| C2 | Measure 2.1 or 2.2 | *As above* | As above for each measure | As above | A positive outcome if any one of the measures is positive | As above |
| 3.1 | NRS Fatigue last week | *Fatigue* | Measures levels of fatigue | 0-10 numerical rating scale | Lower scores mean less fatigue | Reduction of 30% compared to baseline (24) |
| 3.2 | CIS (25) | *Fatigue* | Measures subjective tiredness | 20 statements scored on a 1-7 scale | A sum score is calculated, ranging from 20 to 140. Lower scores mean less exhaustion | Effect size Cohen > 0.8 (23) |
| C3 | Measure 3.1 or 3.2 | *As above* | As above for each measure | As above | A positive outcome if any one of the measures is positive | As above |
| 4 | GPE Coping | *Coping* | Measures to what degree the patient reports improvement compared to the start of the treatment with regard to coping with the consequences of the pain or the pain itself | 1-7 global perceived effect scale | Lower scores mean more improvement | Patients with scores 1, 2 or 3 are considered responders (20) |
| 5 | SF-12 PCS (26) | *Health-related quality of life Physical Component Score* | Measures general health status in 8 domains (physical functioning, role limitations due to physical problems, bodily pain, general health, vitality, social functioning, role limitations due to emotional problems, and mental health) | 12 items, 10 of which are scored on a 1-5 scale and 2 on a 1-3 scale | A Physical (PCS) component summary are calculated by differentially weighing the answers. The summary score ranges from 0 to 100, higher scores mean better physical health | Effect size Cohen > 0.8 (23) |

PDI = Pain Disability Index, GPE = General Perceived Effect, PCS =Patient-Specific Complaints, NRS = Numeric Rating Scale, CIS = Checklist Individual Strength, SF-12 PCS = Short Form quality of life survey Physical Component Score. GPE <3 includes score of totally or much improved, GPE < 4 includes score of totally, much or somewhat improved.
