# Supplementary material for: Machine learning clinical decision support for interdisciplinary multimodal chronic musculoskeletal pain treatment
Source: Front Pain Res (Lausanne). 2023 May 9;4:1177070. doi: 10.3389/fpain.2023.1177070 (PMC10203229; doi:10.3389/fpain.2023.1177070)
Supplement: Supplementary file 5 [file Table5.docx]

Supplementary material E: Algorithm results and validation by outcome (shaded columns)

|  | Endpoint algorithm | % Positive | AUC | Accuracy | Recall  TPR | Specificity  TNR | Precision  PPV | F1 score | Algorithm % positive | Algorithm % negative |
| --- | --- | --- | --- | --- | --- | --- | --- | --- | --- | --- |
| 1.1 | PDI (ENS-SD) | 58.2 | 0.63 | 0.61 | 0.89 | 0.23 | 0.61 | 0.73 | 65.7 | 34.3 |
| 1.2 | GPE disability <4 (ENS-BT) | 84.9 | 0.53 | 0.85 | 1.00 | n/a | 0.85 | 0.92 | 100.0 | 0 |
| 1.2a | GPE disability <3 (SVM-L) | 39.7 | 0.65 | 0.63 | 0.32 | 0.83 | 0.56 | 0.41 | 13.3 | 86.7 |
| 1.3 | PSK 2020 (SVM-CG) | 70.1 | 0.49 | 0.70 | 1.00 | n/a | 0.70 | 0.82 | 94.7 | 5.3 |
| C1 | 1.1 or 1.2 or 1.3 (ENS-SD) | 84.9 | 0.64 | 0.85 | 1.00 | n/a | 0.85 | 0.92 | 90.4 | 9.6 |
| 2.1 | GPE pain (ENS-BT) | 68.7 | 0.62 | 0.69 | 0.97 | 0.09 | 0.70 | 0.81 | 95.6 | 4.4 |
| 2.2 | Pain NRS AVP3 (LR) | 42.4 | 0.65 | 0.62 | 0.37 | 0.81 | 0.59 | 0.45 | 19.0 | 81.0 |
| C2 | 2.1 or 2.2 (ENS-BT) | 72.6 | 0.60 | 0.73 | 0.98 | 0.06 | 0.73 | 0.84 | 89.0 | 11.0 |
| 3.1 | Fatigue NRS AVP5 (ENS-SD) | 36.3 | 0.61 | 0.64 | 0.09 | 0.96 | 0.56 | 0.15 | 16.5 | 83.5 |
| 3.2 | CIS total (ENS-SD) | 52.3 | 0.60 | 0.57 | 0.72 | 0.42 | 0.57 | 0.64 | 57.0 | 43.0 |
| C3 | 3.1 or 3.2 (SVM-FG) | 61.5 | 0.52 | 0.61 | 1.00 | n/a | 0.61 | 0.76 | 85.7 | 14.3 |
| 4 | GPE coping (LR) | 86.3 | 0.64 | 0.86 | 1.00 | 0.02 | 0.86 | 0.93 | 97.9 | 2.1 |
| 5 | SF12 PCS (KNB) | 59.7 | 0.58 | 0.60 | 0.94 | 0.10 | 0.61 | 0.74 | 73.9 | 26.1 |

N=2,364 as algorithms provide estimates for all endpoints
