# Supplementary figures and images for: Machine learning clinical decision support for interdisciplinary multimodal chronic musculoskeletal pain treatment
Source: Front Pain Res (Lausanne). 2023 May 9;4:1177070. doi: 10.3389/fpain.2023.1177070 (PMC10203229; doi:10.3389/fpain.2023.1177070)

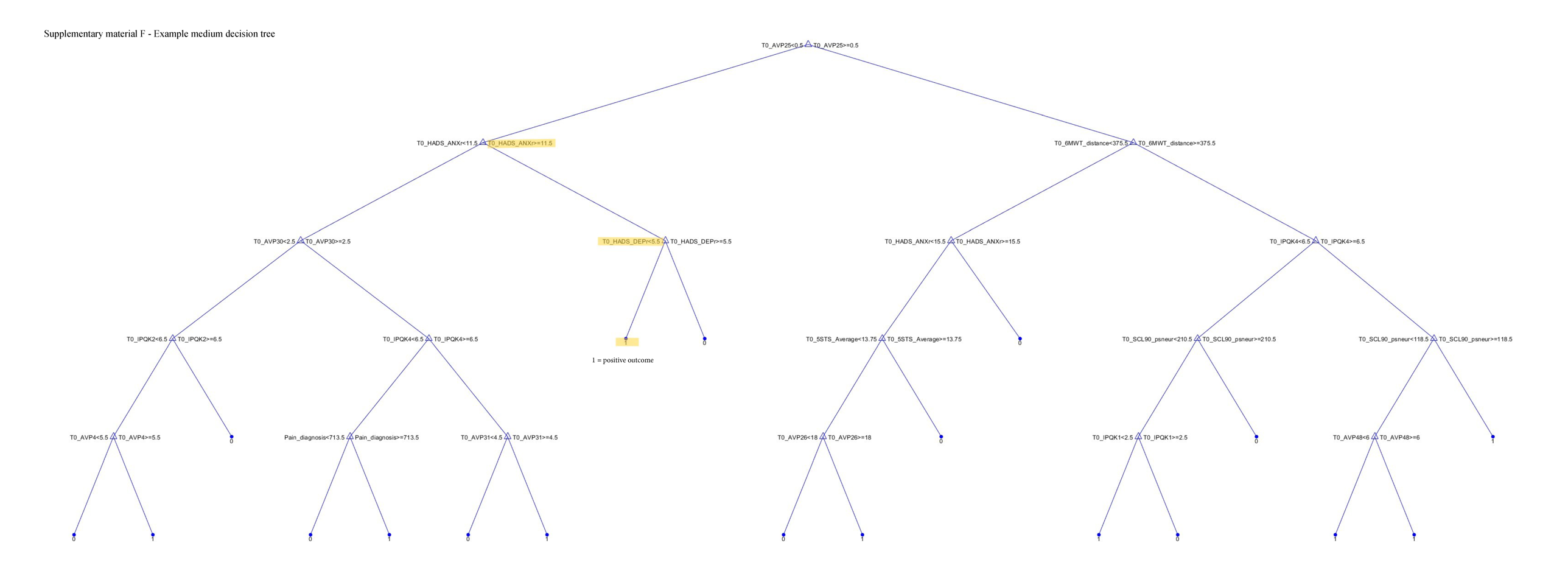

Supplement: Supplementary file 6 [file Image1.tiff]
